# Repeatability and Reproducibility Uncertainty in Magnetic Resonance-Based Electric Properties Tomography of a Homogeneous Phantom

**DOI:** 10.3390/tomography9010034

**Published:** 2023-02-17

**Authors:** Alessandro Arduino, Francesca Pennecchi, Ulrich Katscher, Maurice Cox, Luca Zilberti

**Affiliations:** 1 Istituto Nazionale di Ricerca Metrologica, Strada delle Cacce 91, 10135 Torino, Italy; 2 Philips Research Laboratories, 22335 Hamburg, Germany; 3 National Physical Laboratory, Teddington TW11 0LW, UK

**Keywords:** electric properties tomography, magnetic resonance imaging, phantoms, quantitative imaging, uncertainty

## Abstract

Uncertainty assessment is a fundamental step in quantitative magnetic resonance imaging because it makes comparable, in a strict metrological sense, the results of different scans, for example during a longitudinal study. Magnetic resonance-based electric properties tomography (EPT) is a quantitative imaging technique that retrieves, non-invasively, a map of the electric properties inside a human body. Although EPT has been used in some early clinical studies, a rigorous experimental assessment of the associated uncertainty has not yet been performed. This paper aims at evaluating the repeatability and reproducibility uncertainties in phase-based Helmholtz-EPT applied on homogeneous phantom data acquired with a clinical 3 T scanner. The law of propagation of uncertainty is used to evaluate the uncertainty in the estimated conductivity values starting from the uncertainty in the acquired scans, which is quantified through a robust James–Stein shrinkage estimator to deal with the dimensionality of the problem. Repeatable errors are detected in the estimated conductivity maps and are quantified for various values of the tunable parameters of the EPT implementation. The spatial dispersion of the estimated electric conductivity maps is found to be a good approximation of the reproducibility uncertainty, evaluated by changing the position of the phantom after each scan. The results underpin the use of the average conductivity (calculated by weighting the local conductivity values by their uncertainty and taking into account the spatial correlation) as an estimate of the conductivity of the homogeneous phantom.

## 1. Introduction

Different from traditional magnetic resonance imaging (MRI), which provides weighted images of the relaxation times T1 and T2, quantitative MRI aims at producing images in which each pixel represents the actual measurement of a physical parameter [1]. What makes the change in paradigm from traditional to quantitative MRI particularly appealing is the fact that it is possible to compare objectively the measurements performed during longitudinal studies. In such studies, the evolution of a disease and the efficacy of therapy are monitored by scanning the patient after a certain amount of time [2]. Moreover, the objectivity of a quantitative MRI scan could allow the adoption of reliable automatic systems for image analysis and the identification of new quantitative imaging biomarkers.

Amongst the physical quantities that can be measured with an MRI scanner, there are the electric properties of the biological tissues [3]. It has been observed that the value of the electric properties, in particular of the electric conductivity, in certain pathologies (breast cancer [4], glioma [5], fibrotic liver [6], cerebral ischemia [7]) is different than in the corresponding healthy tissues. Moreover, for therapies based on the use of electromagnetic fields, the knowledge of the actual distribution of the electric properties within the patient body allows implementing a form of patient-specific precision medicine, through personalized treatment planning [8].

Magnetic resonance-based electric properties tomography (EPT) is a quantitative MRI technique that provides a map of the distribution of electric conductivity and permittivity within the imaged subject [3,9,10]. The dispersive electric properties of biological tissues [11] are estimated at the Larmor frequency of the MRI scanner by processing the spatial distribution of the radiofrequency (RF) magnetic flux density $B_{1}$ transmitted by the transmit RF coil of the scanner. Specifically, the transmitted complex magnetic flux density component $B_{1}^{+}$ that rotates in the same direction as the spin precession [12] can be measured by the MRI scanner. Its magnitude, called transmit sensitivity, is estimated through B1 mapping techniques [13,14,15,16], which, in general, are time-consuming and not routinely included in clinical studies. The possibility of performing B1 mapping through magnetic resonance fingerprinting [17], which is known for its short scanning time, could increase the availability of transmit sensitivity acquisitions in clinical setups in the near future. The phase is properly approximated, in 1.5 T or 3 T scanners, by half the transceive phase $\phi^{\pm }$, acquired as the phase of suitable, fast conventional MRI sequences [9]. Under special symmetry conditions, the same conventional MRI sequence, applied with a low flip-angle [18], can also provide an estimate of the transmit sensitivity.

To obtain the map of the electric properties, $B_{1}^{+}$ can be processed in different ways according to a plethora of EPT implementations proposed in the literature [10]. Their development is similar to that of the well-known microwave imaging techniques [19,20,21]. The similarity appears clear, especially with the EPT methods based on optimization theory [22,23,24,25,26,27,28,29,30,31,32]. Nonetheless, knowledge of the value of $B_{1}^{+}$ at a large number of points within the inspected body suggests that EPT can achieve a larger effective resolution than traditional microwave imaging [19,25].

The EPT implementation more commonly adopted in early clinical studies [4,5,6,33] is phase-based Helmholtz-EPT (HH-EPT) [34,35,36], which estimates only the electric conductivity by processing the transceive phase $\phi^{\pm }$. This EPT approach is convenient because it is based on a simple, linear and real-valued algebraic equation and relies on a routine MRI sequence for $\phi^{\pm }$ acquisition, which is fast enough to allow monitoring of dynamical processes [37] or imaging regions where motion occurs, such as the cardiac region [38]. On the other hand, HH-EPT suffers from large systematic errors at the internal boundaries between different biological tissues, where the electric properties change abruptly [39,40]. The quality of the recovered maps can be improved by post-processing, for example, with a median filter, according to the subject anatomy delineated by the acquired MRI scans [5].

In order to make the result of a quantitative MRI technique a proper measurement result in the strict sense, the uncertainty with which the property values have been estimated must be assessed [41]. Up to now, only a few works have dealt with uncertainty quantification in EPT and only in theoretical cases [42,43]. In this paper, the uncertainty of the results of phase-based HH-EPT applied to a homogeneous phantom acquired by a 3 T MRI scanner with a routine three-dimensional sequence is assessed under repeatability and reproducibility conditions. The former condition is obtained by arranging the phantom in the scanner and acquiring it repeatedly with the same pulse sequence; the latter is obtained by rearranging the phantom in a different position after each acquisition. Under both conditions, the uncertainty in the three-dimensional estimation of the phantom conductivity is evaluated taking into account the possible correlations between the values estimated in different voxels. To deal properly with the dimensionality of the problem, only a spherical region of radius 3 cm centered at the phantom barycenter is studied. In this way, it is certain that the boundary errors, typical of HH-EPT applied to heterogeneous domains [39,40], are not present in the investigated homogeneous region. Thus, the uncertainty contribution corresponding to the boundary errors is prevented from influencing the results, allowing to evaluate exclusively the uncertainty in the application of HH-EPT to a bulk homogeneous region. Moreover, the uncertainty, described by the covariance matrix of the multivariate random variable modeling the conductivity measurement, is computed according to a robust James–Stein shrinkage estimator [44,45].

The evaluated uncertainty is finally propagated through the estimation of the average conductivity of the phantom and used to improve the result, in terms of both accuracy and precision, with a generalized weighted averaging [46] of the conductivity map. Thus, in addition to assessing the uncertainty of EPT results for a homogeneous phantom under repeatability and reproducibility conditions, this work legitimizes the use of local spatial averages in homogeneous regions, for a quantitative improvement of the recovered maps. As a by-product, a relation between the uncertainty and the spatial dispersion of the estimated conductivity values, useful for practical uncertainty estimation in homogeneous regions, has been identified.

## 2. Materials and Methods

### 2.1. Phantom

A homogeneous cylindrical phantom (volume 2 L, radius ~6 cm, length ~18 cm) filled with a solution of 64 mM of NaCl in distilled water is used for the analysis under repeatability conditions. Recording shows that the temperature of the room with the MRI scanner oscillates between 16 °C and 18 °C. Hence, according to Stogryn’s equations [47], which are well approximated by a linear law in this range of temperature, the electric conductivity of the phantom at 128 MHz ranges from 550 mS m^−1^ to 570 mS m^−1^ and is assumed to be equal to 560 mS m^−1^ ± 10 mS m^−1^. A direct characterization of the phantom conductivity has not been performed. In the following, the expected conductivity value is denoted by $\sigma_{\exp}=$ 560 mS m^−1^.

A replica of this phantom, with a similar but not identical conductivity value, has been adopted for the analysis under reproducibility conditions. Although it is impossible to compare the conductivity values estimated under repeatability and reproducibility conditions in terms of averages, the comparison is still possible regarding the spatial features of the recovered maps and their uncertainties.

### 2.2. MRI Acquisitions

The MRI scanner used for the experiments is a 3 T Ingenia TX (Philips Healthcare, Best, The Netherlands) equipped with a 32-channel RF receive head coil. The scans are acquired with a three-dimensional steady-state free precession (SSFP) sequence with nominal flip-angle of 30° and isotropic resolution of 2 mm. The received signals are processed with the vendor’s algorithm CLEAR [48], so that, from the practical point of view, the system receive sensitivity becomes equal to that of the body-coil used in transmission with switched polarization. The phase of the acquired complex images is a good estimate of the transceive phase $\phi^{\pm }$ [9]. Because of the cylindrical symmetry of the adopted phantom and the low flip-angle of the sequence, the magnitude of the acquired complex images is a good estimate of the square of the transmit sensitivity ${\left|B_{1}^{+}\right|}^{2}$ when the phantom is positioned at the scanner’s isocenter [18].

Twenty-five images are acquired on the phantom centered at the scanner isocenter, without moving the phantom between scans. The acquisitions are performed in groups of five with a break of a few minutes between the groups. Each scan takes one minute. The specific absorption rate of each group has been estimated to induce an increase in the phantom temperature of about 0.05 °C under adiabatic conditions, with a corresponding variation in the electric conductivity of about 0.5 mS m^−1^, well below the 10 mS m^−1^ standard uncertainty with which the expected conductivity is known. We refer to these images as the acquisitions under repeatability conditions, or simply as “repetitions”.

**Figure 1:**
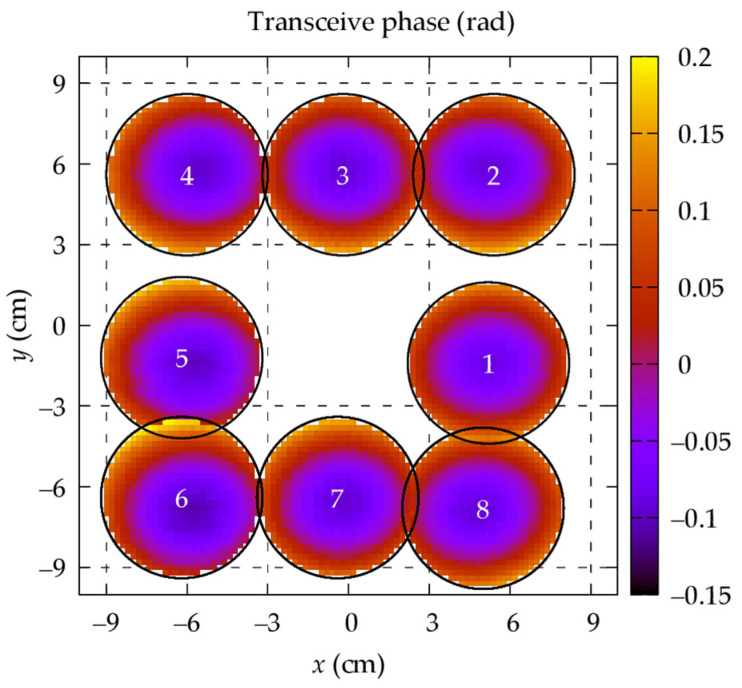
Transceive phase in the scanner mid-plane acquired by moving the phantom around the isocenter under reproducibility conditions. The maps are restricted to a sphere with radius of 3 cm centered at the phantom barycenter.

Eight additional images are acquired by moving the phantom to eight positions around the scanner isocenter, with successive phantom centers about 6 cm apart, as depicted in Figure 1. We refer to these images as the acquisitions under reproducibility conditions, or simply as “reproductions”.

A subtle aspect of the following analysis concerns the way in which the conductivity values can be averaged. When conductivity values belonging to the same map are averaged (i.e., the averaging involves voxels with different locations within the target region), the result is indicated as “average”. Instead, when the averaging is performed between different maps (voxel by voxel, across the repetitions/reproductions), the result is indicated as “mean”.

### 2.3. Phase-Based Helmholtz-EPT

#### 2.3.1. Formulation

In order to estimate the distribution of the electric conductivity σ starting from a map of the transceive phase $\phi^{\pm }$, Maxwell’s equations in the frequency domain are inverted. HH-EPT operates with the assumption that the electric properties are spatially homogeneous [3,9,10]. In this case, Maxwell’s equations can be combined to obtain the vectorial Helmholtz’s equation for the magnetic flux density, which, in scalar form, holds also for its rotating component $B_{1}^{+}$. The latter equation is then inverted algebraically to obtain the fundamental relation of HH-EPT,

(1) $$\epsilon -i\frac{\sigma }{\omega }=-\frac{\nabla^{2}B_{1}^{+}}{\omega^{2}\mu_{0}B_{1}^{+}},$$

where i is the imaginary unit, ϵ the electric permittivity, $\mu_{0}$ the magnetic permeability of vacuum, and ω=2πf the angular frequency of the electromagnetic radiation, with Larmor frequency f= 128 MHz for a 3 T scanner.

By distinguishing magnitude and phase of $B_{1}^{+}=\left|B_{1}^{+}\right|e^{i\phi^{+}}$ in (1), the conductivity is written as:

(2) $$\sigma =\frac{\nabla^{2}\phi^{+}}{\omega \mu_{0}}+\frac{2\nabla \left|B_{1}^{+}\right|\cdot \nabla \phi^{+}}{\omega \mu_{0}\left|B_{1}^{+}\right|}.$$

When the transmit sensitivity $\left|B_{1}^{+}\right|$ is sufficiently homogeneous, the right-hand side of the latter equation is approximated by neglecting the second addend. Since for the adopted scanner $\phi^{+}≅\phi^{\pm }/2$ [9], the following relation, called phase-based HH-EPT, is obtained,

(3) $$\sigma ≅\frac{\nabla^{2}\phi^{\pm }}{2\omega \mu_{0}}.$$

#### 2.3.2. Implementation

Phase-based HH-EPT consists of the application of a second-order linear differential operator, ${\left(2\omega \mu_{0}\right)}^{-1}\nabla^{2}$, to the acquired transceive phase map. Since the acquired maps are discrete and noisy, the differential operator is approximated by a discrete counterpart employing the robust Savitzky–Golay (SG) filter [49]. The SG filter consists in approximating locally the distribution of $\phi^{\pm }$ with a paraboloid, whose Laplacian is then evaluated analytically. Let $p\in {\mathbb{R}}^{N_{p}}$ and $s\in {\mathbb{R}}^{N_{s}}$ denote the vectors that define the discrete three-dimensional maps of $\phi^{\pm }$ and σ, respectively; the application of the SG filter can be represented as a matrix–vector product

(4) s=Ap,

where $A\in {\mathbb{R}}^{N_{s},N_{p}}$ approximates the differential operator.

It is worth underlining that $N_{p}$ is the number of voxels in which the phase measurement is used to retrieve the electric conductivity. $N_{s}$ is the number of voxels where the electric conductivity is retrieved. Precisely, the vectors p and s list the values that the corresponding physical quantities assume in each voxel according to a certain ordering of the voxels. In general, $N_{s}<N_{p}$, because EPT is not performed if the SG filter kernel partially falls outside the investigated domain. Hence, the region where the electric conductivity is estimated changes with the shape and the size of the SG kernel.

Since the SG filter is a local approximation of the differential operator, matrix A is sparse and each row contains non-null values only in the components corresponding to voxels that belong to the filter kernel centered in the voxel associated with the row. In practice, it is unnecessary to build explicitly matrix A, since the SG filter can be applied in a computationally efficient way according to a moving window algorithm. However, the matrix formalism is useful for propagating the covariance matrix as described below.

**Figure 2:**
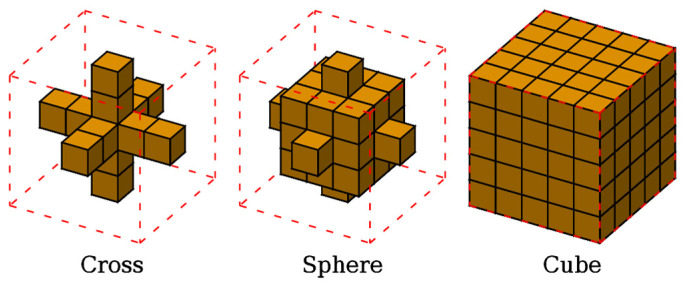
Kernels of the Savitzky–Golay filter with different shapes for n=2, where n indicates the number of voxels placed along the Cartesian directions on each of the six sides of the central voxel. The dashed red lines depict the cubes bounding the kernels.

In the following, the electric conductivity is estimated by applying the SG filter with different kernels to check their influence on the quality of the result. Three kernel shapes are considered: a cross, a voxelized sphere, and a cube. For each shape, five sizes of the cube bounding the kernel are analyzed, with edges equal to 2n+1 voxel widths for n=1,…,5. Figure 2 sketches the kernel shapes for n=2. The number of voxels involved in the Laplacian estimation varies with the shape and size of the kernel.

The phase-based HH-EPT implementation provided in EPTlib 0.1.1 [36] is adopted here.

### 2.4. Covariance Matrix

The covariance matrix is the mathematical way to express the fact that different variables are interdependent, providing a quantification of their correlation. The EPT problem under study is a multivariate measurement (i.e., a measurement with multiple output quantities, represented by the conductivity values of the different voxels) described by Equation (4). In this case, the output conductivity values in vector s exhibit covariance because of two different reasons, ascribable to matrix A and vector p, respectively. First, matrix A is not diagonal. This means that the conductivity in a given voxel is obtained by elaborating the values of the phase measured within the corresponding kernel, some of which are used also in other kernels (to estimate the conductivity of other voxels). Hence, the same input influences different outputs with the result that correlation exists between outputs that share the same inputs. However, the correlation in the output s may be also partly inherited from the correlation already present in the input p. The latter originates from the fact that the phase values depend, in turn, on even more fundamental quantities, such as the details of the measurement pipeline adopted by the MRI scanner to acquire them. Common dependence on those quantities induces correlation in the phase values. Thus, a non-diagonal covariance matrix can also be associated with variable p.

The evaluation of the covariance matrix is the fundamental step in uncertainty assessment [50]. The covariance matrix of the estimated distribution of electric conductivity, denoted by $\Sigma \left(s\right)\in {\mathbb{R}}^{N_{s},N_{s}}$, encompasses all the information regarding the measurement uncertainty and depends on the adopted SG kernel shape and size. The i-th diagonal term $\Sigma {\left(s\right)}_{i,i}$ of the covariance matrix contains the variance of the value estimated for electric conductivity in the i-th voxel; the off-diagonal term $\Sigma {\left(s\right)}_{i,j}$ contains the covariance between the values estimated in the i-th and in the j-th voxels.

The covariance matrix is a symmetric and positive definite matrix and, in general, it is dense. This makes its evaluation particularly demanding, both from the computational point of view (because a large dense matrix occupies a great amount of memory) and from the experimental point of view (because many data are needed for an accurate evaluation of all the matrix entries).

In the present investigation, few data are available (25 acquisitions under repeatability conditions and 8 acquisitions under reproducibility conditions), whereas the covariance matrix is a very large square matrix of order equal to the number of voxels. In order to reduce the dimensionality of the problem, only the conductivity values retrieved from the phase acquired in a sphere with radius of 3 cm, centered at the phantom barycenter, are analyzed. In the following, this sphere is denoted by $B_{p}$. This leads to $N_{p}≅11,500$ and $N_{s}$ varying from ∼9 600 for the cross-shaped SG kernel with n=1 to ∼900 for the cubic SG kernel with n=5. Please note that this is not the size of the kernel, which is 7 voxels for the cross-shaped SG kernel with n=1 and 1 331 voxels for the cubic SG kernel with n=5.

By limiting the investigation to the sphere $B_{p}$, the uncertainty contributions in the conductivity maps estimated by HH-EPT due to boundary errors are avoided. This results in evaluating exclusively the uncertainty in the application of HH-EPT to a bulk homogeneous region.

Two approaches are followed in the evaluation of the covariance matrix, depending on the measurement conditions.

#### 2.4.1. Repeatability Conditions

Under repeatability conditions, since the phantom is not moved from one scan to another, the acquired phase maps always describe the very same phase distribution. The observed differences between maps are related to the measurement uncertainty, which can be quantified through the covariance matrix of the mean phase distribution, denoted by $\Sigma \left(p\right)\in {\mathbb{R}}^{N_{p},N_{p}}$.

To avoid spurious correlations due to constant differences between the acquired phase maps, which could appear but do not affect the Laplacian in (3), all the phase maps are translated to guarantee a null spatial average within the sphere $B_{p}$. The mean of the resulting set of maps is calculated, voxel-by-voxel, to provide the mean phase distribution p, from which the estimated conductivity distribution s is computed.

The experimental covariance matrix [50] S(p) is computed starting from the 25 repetitions $p_{k}$, with k=1,…,25, as

(5) $$S\left(p\right)=\frac{1}{n\left(n-1\right)}\overset{25}{\underset{k=1}{\sum }}\left(p_{k}-p\right){\left(p_{k}-p\right)}^{T}.$$

Since twenty-five acquisitions are a small number with respect to the number of voxels, the experimental covariance matrix S(p) might provide a poor estimate of the actual covariance matrix Σ(p). Indeed, it could lead to strong misleading correlations. A better estimator is provided by the James–Stein shrinkage [44,45], which shrinks the experimental matrix towards its diagonal by combining in an optimal way the experimental matrix defined by the acquired data with a conjectured diagonal matrix. This results in

(6) Σ(p)≅λS(p)+(1−λ)diag(S(p)),

where diag(⋅) denotes the diagonal matrix having the same diagonal of its argument and λ∈[0,1] is determined analytically as the coefficient that minimizes the Frobenius norm of the difference between the actual and the estimated covariance matrices [44,45].

Finally, the estimated Σ(p) is propagated through the linear model (4) according to the law of propagation of uncertainty [50,51], leading to

(7) $$\Sigma \left(s\right)=A\Sigma \left(p\right)A^{T}.$$

For computational efficiency, the propagation has been evaluated taking advantage of the features of the SG filter without building matrix A explicitly. The code used is available in [52].

#### 2.4.2. Reproducibility Conditions

Under reproducibility conditions, since the phantom is moved from one scan to another, the acquired phase maps describe different phase distributions. This change makes it physically unreasonable to calculate the mean of the phase maps and to evaluate their measurement uncertainty through the covariance matrix of the mean. Hence, for each phase distribution $p_{k}$ acquired under reproducibility conditions (with k=1,…,8), the corresponding conductivity distribution $s_{k}$ is computed with (4).

The homogeneous phantom keeps the same electric conductivity when rearranged within the scanner. Thus, all the obtained conductivity maps describe the same distribution, and their mean can be calculated, voxel-by-voxel, to obtain the mean conductivity distribution s, whose covariance matrix quantifies the measurement uncertainty. Note that, when moving the phantom, the correspondence between the material and geometric voxels is not strictly maintained (because of the possible movement of the saline solution inside the phantom). However, since the phantom is homogeneous, the acquired phase distribution is unaffected by the material rearrangement that takes place inside the phantom itself. The phase distribution is exclusively affected by the macroscopic positioning of the entire phantom within the scanner and by the ubiquitous random effects due to system and measurement imperfections.

By considering only the phase values retrieved in the sphere $B_{p}$, it is guaranteed that analogous geometrical regions are compared between the different reproduced acquisitions. Additionally, in this case, the number of acquisitions is very small with respect to the number of voxels, leading to an unsuitable experimental covariance matrix. Thus, Σ(s) is computed again through the James–Stein shrinkage estimator described above.

### 2.5. Spatial Averaging

Since the phantom is homogeneous, its electric conductivity distribution can be described by a single value, estimated by the spatial average of the measured conductivity map,

(8) $$\bar{s}=\frac{1}{N_{s}}\overset{N_{s}}{\underset{i=1}{\sum }}s_{i},$$

where $s_{i}$ denotes the i-th component of s. The standard uncertainty of $\bar{s}$ is quantified by propagating the covariance matrix through its analytical expression [50], namely

(9) $$u\left(\bar{s}\right)=\frac{1}{N_{s}}\sqrt{\overset{N_{s}}{\underset{i=1}{\sum }}\overset{N_{s}}{\underset{j=1}{\sum }}\Sigma {\left(s\right)}_{i,j}},\;\;\;\;\;u_{r}\left(\bar{s}\right)=\frac{u\left(\bar{s}\right)}{\bar{s}},$$

where $u_{r}\left(\bar{s}\right)$ is the relative uncertainty of $\bar{s}$.

A better estimator of the spatial average takes into account the uncertainty associated with the measured conductivity distribution by weighting the vector s with the precision matrix (i.e., the inverse of the covariance matrix) [46]. Thus, the lower the uncertainty of the conductivity value in a given voxel, the more it contributes to the weighted average. This leads to the equation

(10) $${\bar{s}}_{w}=\frac{1^{T}\Sigma {\left(s\right)}^{-1}s}{1^{T}\Sigma {\left(s\right)}^{-1}1}\;,$$

where $1\in {\mathbb{R}}^{N_{s}}$ denotes a column vector of ones. It is worth noting that the latter equation reduces to (8) if the identity matrix is used in place of the precision matrix. The law of propagation of uncertainty [50] applied to the weighted average allows the uncertainty of ${\bar{s}}_{w}$ according to [46] to be quantified:

(11) $$u\left({\bar{s}}_{w}\right)=\frac{1}{\sqrt{1^{T}\Sigma {\left(s\right)}^{-1}1}},\;\;\;\;\;u_{r}\left({\bar{s}}_{w}\right)=\frac{u\left({\bar{s}}_{w}\right)}{{\bar{s}}_{w}},$$

$u_{r}\left({\bar{s}}_{w}\right)$ being the relative uncertainty of ${\bar{s}}_{w}$.

## 3. Results

### 3.1. Repeatability Conditions

**Figure 3:**
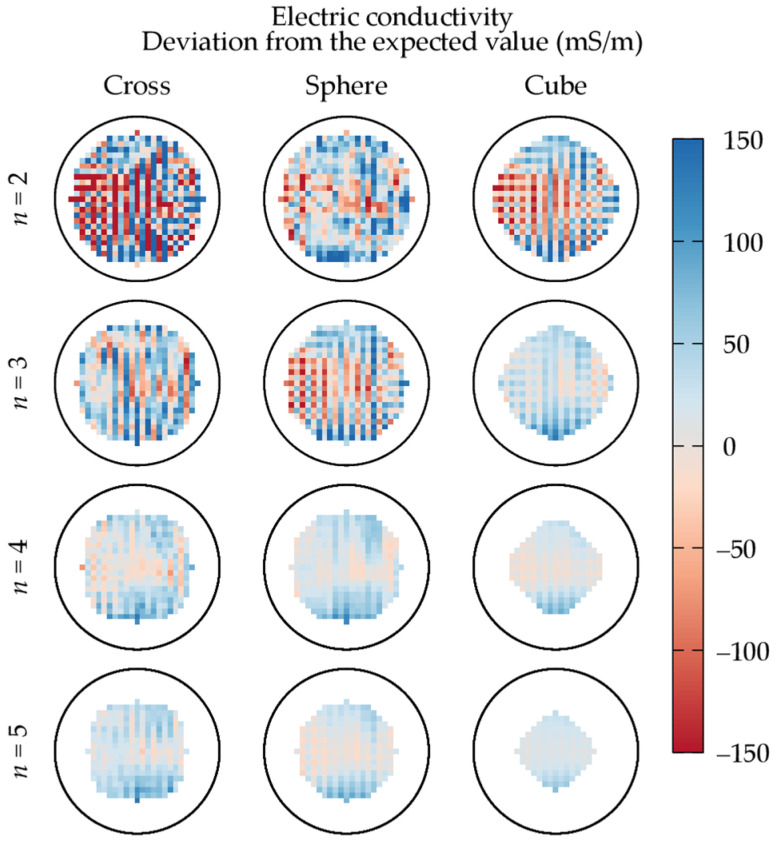
Relative deviation from the expected value ($\sigma_{\exp}=$ 560 mS m^−1^) of the mean electric conductivity estimated in the scanner mid-plane by phase-based Helmholtz-EPT under repeatability conditions. The results are reported for different shapes and sizes of the kernel of the Savitzky–Golay filter. The black circle denotes the section of the sphere $B_{p}$.

Despite the phantom homogeneity, the mean electric conductivity maps estimated by applying phase-based HH-EPT to the acquired repetitions are not homogeneous. The estimations are characterized by a spatial noise that makes the conductivity range around the expected value $\sigma_{\exp}$. The relative deviations from $\sigma_{\exp}$ are shown in Figure 3 in the scanner mid-plane for different shapes and sizes of the SG kernel. It is worth noting that, since the number $N_{p}$ of voxels whose transceive phase value is used is constant (equal to the number of voxels belonging to the sphere $B_{p}$), the number $N_{s}$ of voxels where the electric conductivity is estimated becomes smaller when the SG kernel becomes larger.

From Figure 3, it is seen that the spatial noise in the phase-based reconstruction is lower when the volume of the filter kernel is larger. In particular, the conductivity estimated with the largest cubic kernel appears almost homogeneous when compared with the estimations obtained with smaller kernels. Moreover, the deviation from $\sigma_{\exp}$ reported in Figure 3 is mainly positive for the largest cubic kernel, suggesting an overall overestimation of the electric conductivity by phase-based HH-EPT. This deviation is in accordance with the observations about the systematic error introduced by the phase-based approximation reported in a previous publication [34], i.e., the second addend of (2) is negative in most cases.

For all the considered kernels, the propagated covariance matrix Σ(s) shows a typical voxel-wise uncertainty sensibly lower than the spatial dispersion of the estimated conductivity map (i.e., the standard deviation of the spatial distribution). For example, using the spherical kernel with n=3, the repeatability uncertainty in each voxel is evaluated around 13 mS m^−1^, whereas the spatial dispersion is equal to 68 mS m^−1^. Indeed, some artifacts affect the phase maps leading to repeatable systematic errors in the EPT result that appear similar to random noise, but that are not canceled out by taking the mean map of multiple acquisitions.

**Figure 4:**
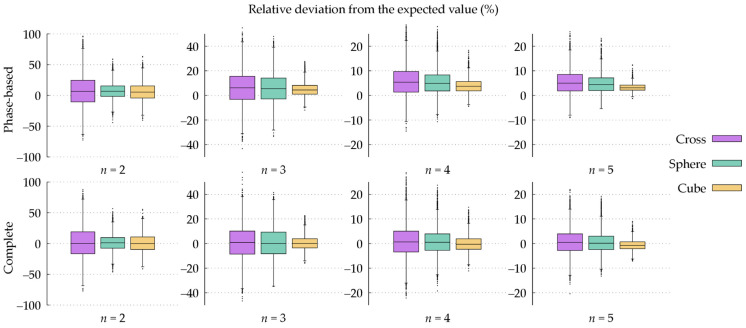
Boxplots of the relative deviation from the expected value ($\sigma_{\exp}=$ 560 mS m^−1^) of the mean electric conductivity maps estimated by Helmholtz-EPT under repeatability conditions when different shapes and sizes of the kernel of the Savitzky–Golay filter are used. The top row shows the results of the phase-based method (3), whereas the bottom row shows those of the complete method (1).

A statistical insight into the spatial dispersion of the estimated electric conductivity maps is provided by the boxplots collected in Figure 4. The estimated conductivity values are always distributed symmetrically, independently of the kernel adopted for the SG filter. Moreover, the median value, corresponding to the spatial average for a symmetric distribution, is almost the same for all the considered shapes and sizes of the filter kernel. A slightly lower value is obtained only with the largest cubic kernels. As already observed above, the dispersion of the estimated values, quantifiable through the interquartile range reported by the boxplots, is lower when the volume of the filter kernel is larger.

Figure 4 reports the boxplots of the electric conductivity values estimated with both the phase-based HH-EPT (3) and the complete HH-EPT (1). This is possible because the simple geometry of the phantom and its central positioning with respect to the RF coils allows the transmit sensitivity $\left|B_{1}^{+}\right|$ from the acquired images to be estimated. The comparison between the boxplots produced with the two EPT methods shows that the median value estimated with the complete HH-EPT deviates very little from $\sigma_{\exp}$. However, the complete HH-EPT result retains the statistical dispersion of the values estimated with phase-based HH-EPT. The correction term in (2), where the transmit sensitivity appears, is small in magnitude with respect to the Laplacian of the phase. Moreover, it involves only first-order derivatives, which can be estimated with less noise propagation than the Laplacian. Thus, despite the presence of an additional noise contribution in the estimation of the conductivity values due to the measured transmit sensitivity, a larger dispersion in the case of complete HH-EPT with respect to the phase-based HH-EPT was not expected.

**Figure 5:**
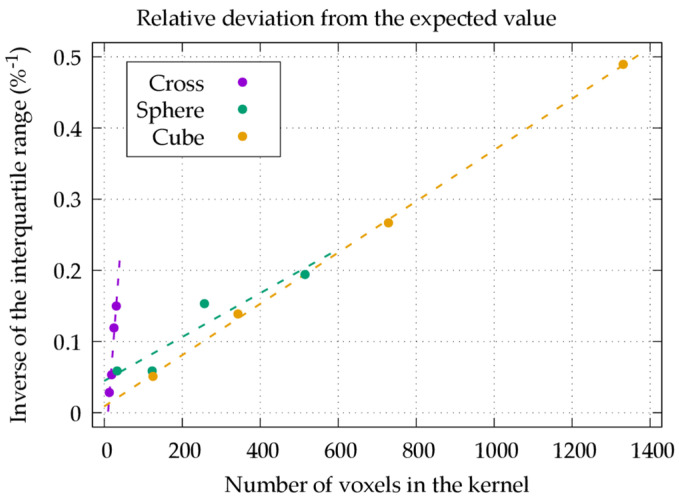
Trend of the inverse of the dispersion, quantified through the interquartile range, of the mean electric conductivity values estimated with the phase-based Helmholtz-EPT under repeatability conditions with respect to the number of voxels in the kernel of the Savitzky–Golay filter.

Since the spatial dispersion of the estimated conductivity maps can be reduced by enlarging the SG kernel, it could be expected that each single voxel provides an additional amount of information to the SG filter for compensating the input artifacts. Figure 5 shows that the amount of information contributed by each voxel depends on the shape of the kernel, i.e., on the relative positioning of the voxels. Specifically, when the number of voxels in the kernel is increased by increasing parameter n is increased, the cross-shaped kernel reduces the dispersion significantly faster than the spherical and the cubic kernels in relative terms, although it is penalized by its smaller global number of involved voxels.

The spatial averages of the electric conductivity maps provided by phase-based HH-EPT applied with different shapes and sizes of the SG filter are collected in Table 1. Both the average $\bar{s}$ and the weighted average ${\bar{s}}_{w}$ are reported, each one with its relative standard uncertainty. The uncertainties associated with the spatial averages are very small for all considered kernels in the SG filter. In particular, $u\left(\bar{s}\right)$ is almost always smaller than 1 mS m^−1^ and $u\left({\bar{s}}_{w}\right)$ is even smaller than 0.1 mS m^−1^. This indicates very good repeatability of conductivity estimation for a given SG kernel.

The value of $\bar{s}$ ranges from about 578 mS m^−1^, estimated with the largest cubic SG kernel, to about 605 mS m^−1^, estimated with the smallest cross-shaped SG kernel. The inconsistency of these averaged values is corrected by the weighted average ${\bar{s}}_{w}$, which, in addition to having a lower uncertainty, is also more consistent, showing a value of about 586 mS m^−1^ with all the SG kernels but the largest cubic ones, for which the value drops to about 580 mS m^−1^.

### 3.2. Reproducibility Conditions

The way in which the kernel of the SG filter affects the estimation of the electric conductivity map under reproducibility conditions is analogous to what was discussed above in the analysis under repeatability conditions. In the following, the results obtained with a spherical SG kernel with n=3 are reported as an example. However, the presented comments and discussions held for all the considered SG kernels.

**Figure 6:**
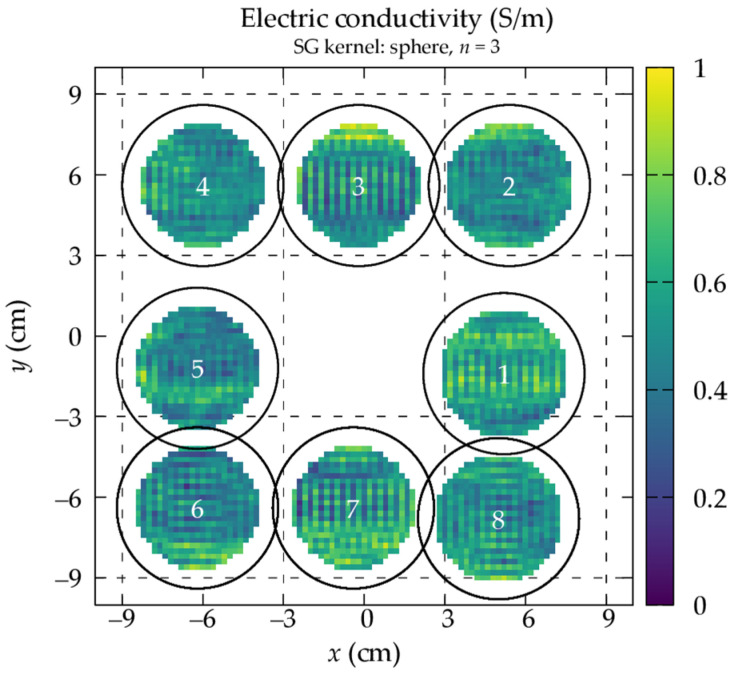
Electric conductivity maps estimated with the phase-based Helmholtz-EPT from the acquisitions under reproducibility conditions. The reported results are obtained with a spherical Savitzky–Golay kernel with n=3.

The conductivity maps reported in Figure 6 are obtained by applying the phase-based HH-EPT to the acquired reproductions (see Figure 1). Each estimated conductivity map is affected by the systematic error due to the phase-based approximation [34], whose magnitude depends on the distribution of the transmit sensitivity $\left|B_{1}^{+}\right|$ within the imaging region. Since moving the phantom in the scanner bore changes the $\left|B_{1}^{+}\right|$ distribution to which the phantom is exposed, each estimated map is affected by this error in a different and non-trivial way. This effect is represented in Figure 6, where some distributions appear brighter (see position 1) and other darker (see position 5).

In addition to the error introduced by the phase-based approximation, the estimated conductivity maps are also affected by the measurement noise and the other artifacts’ contributions whose presence was already observed in the analysis under repeatability conditions. Repositioning the phantom within the scanner changes the spatial distribution of these artifacts, which affect the estimated conductivity values with both positive and negative errors in an unbiased way, as highlighted by the symmetry observed in the boxplots of Figure 4. Although repeatable (i.e., stable if repetitions are performed in a given position), these error contributions appear as mostly random under reproducibility conditions. Thus, they contribute to the reproducibility uncertainty, which results in it inevitably being larger than the repeatability uncertainty. This fact reflects the larger variability in the results of different reproductions with respect to the variability observed for different repetitions.

**Figure 7:**
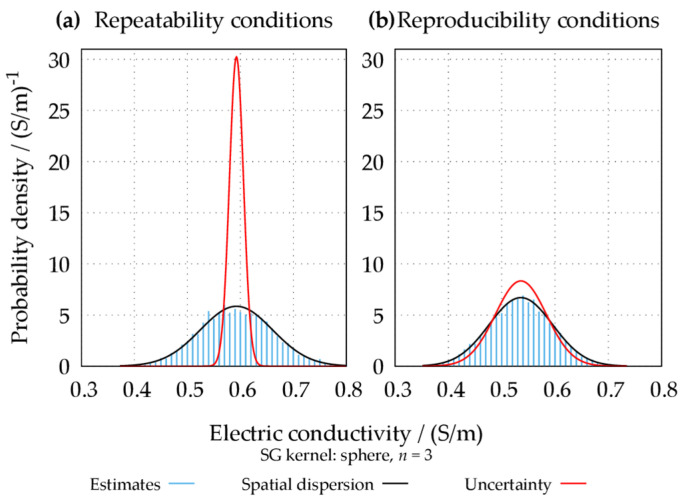
Comparison between the statistical distribution of the estimated mean electric conductivity values, their spatial dispersion, and their average voxel-wise uncertainty. The spatial dispersion is represented through a Gaussian curve with the same mean and standard deviation as the estimates. The uncertainty is represented through a Gaussian curve with the same mean as the estimates and the standard deviation equal to the average of the uncertainties associated with each estimate. The comparison is reported both for repetitions (**a**) and reproductions (**b**).

The non-reproducibility of a significant portion of the observed repeatable artifacts is well quantified by comparing the reproducibility uncertainty deduced from the average of the diagonal values of the propagated covariance matrix Σ(s) with the spatial dispersion of the estimated mean conductivity map (i.e., the standard deviation of the spatial distribution). For the spherical kernel with n=3, the reproducibility uncertainty in each voxel is evaluated to be around 48 mS m^−1^, whereas the spatial dispersion is equal to 60 mS m^−1^. A graphical representation of the discrepancy between the repeatability and reproducibility uncertainties and the spatial dispersion is reported in Figure 7. The figure collects the histograms of the mean electric conductivity values computed in repeatability and reproducibility conditions, evaluated for every single voxel in the target region. Both histograms follow a Gaussian distribution, illustrating that the mean maps computed under both conditions are affected by spatially random noise. Under repeatability conditions, the spatial dispersion is much larger than the average voxel-wise repeatability uncertainty; thus, the spatial noise is repeatable and only apparently random when the position of the phantom does not change. On the other hand, under reproducibility conditions, the reproducibility uncertainty is almost equal to the spatial dispersion; hence, from a practical point of view, the spatial noise is random for reproduced acquisitions. It must be underlined that the uncertainty (i.e., the “width” of the red curves in Figure 7) quantifies the “stability” of the results across different repetitions/reproductions. However, a smaller uncertainty (i.e., higher precision) does not necessarily correspond to better accuracy (i.e., the correct “center” of the curves in Figure 7). With reference to Figure 7, the behavior of each voxel in the target region is more stable across repetitions rather than across reproductions. This is reflected by the lower uncertainty exhibited by repeatability experiments with respect to reproducibility experiments. Nevertheless, this behavior may be affected by systematic biases that are partly disrupted by the change in the phantom position in reproducibility experiments.

## 4. Conclusions

The presented investigation assesses the quality of phase-based HH-EPT applied to retrieve the electric conductivity of a homogeneous phantom under repeatability and reproducibility conditions. The former condition is obtained by acquiring multiple images without moving the sample from one acquisition to another; the latter condition is obtained by changing the position of the phantom within the scanner after each acquisition.

It has been observed that in addition to the systematic error due to the phase-based approximation [34], other repeatable artifacts affect the MRI acquisitions, leading to estimated conductivity maps with a large spatial dispersion that is not described by the small voxel-wise repeatability uncertainty evaluated on the acquired data. However, these repeatable artifacts are not reproducible, and the evaluated voxel-wise reproducibility uncertainty describes almost completely the spatial dispersion observed in the estimated maps. Being unrelated to the phase-based approximation, these observations hold for the complete HH-EPT as well.

One conclusion is that averaging multiple repeated acquisitions cannot improve indefinitely the quality of the estimated conductivity map. Indeed, this effect was already observed for a homogeneous phantom [53] and is now assessed in terms of repeatability uncertainty. Moreover, averaging acquisitions performed whilst rearranging the phantom after each scan reduces the spatial dispersion faster than in the case of repeated acquisitions.

An important aspect deduced from the present analysis is that the reproducibility uncertainty can be directly estimated from the spatial dispersion of a single acquisition. This result holds for the definition of reproduction adopted in this work and should be integrated with the contribution of other sources of uncertainty not considered here, such as the variability introduced by the MRI scanner used or the adopted acquisition sequence, to provide the complete measurement uncertainty. Despite further work being needed to reach a complete uncertainty budget for HH-EPT, it is promising to note that a non-trivial uncertainty contribution like that due to the positioning of the sample within the scanner can be estimated from the output of a single acquisition.

The robustness of the weighted spatial averages in Table 1, as well as the stability of the median values in the boxplots of Figure 4, suggests that the adoption of post-processing strategies based on local filters applied to homogeneous regions, like the ones used in [5], could improve the overall quality of the estimated conductivity maps without losing their quantitative features.

The study has been performed on an MR system built by one of the standard commercial manufacturers. It is expected that the main results of the study, particularly the conclusions drawn from Figure 7, are independent of the specific manufacturer, as they reflect general physical features. Other aspects (e.g., related to SNR and noise pattern) might differ between manufacturers, but investigating these differences was beyond the scope of this study.

It is worth noting that the results presented here have been obtained for a homogeneous phantom and are, hence, rigorously valid only under the same conditions. Precisely, the analysis has been conducted including only voxels in a sphere at the phantom barycenter, far from the phantom boundary, where the boundary artifacts of HH-EPT take place [39,40]. It is likely that the conclusions reached could also be extended to the innermost part of homogeneous compartments of heterogeneous domains, a fundamental step to allow uncertainty assessment in clinical applications.

An analysis similar to the one presented in this paper can be performed also for the permittivity estimated by the complete HH-EPT [3,9,10], although in that case, the fundamental equation is not linear. However, currently, the recovered permittivity maps have a very low SNR and require the introduction of additional sequences to the scanning protocol in order to also acquire the transmit sensitivity, making them less interesting than the conductivity maps provided by phase-based HH-EPT.

## Figures and Tables

**Table 1:** Spatial averages of the conductivity estimated with phase-based Helmholtz-EPT under repeatability conditions.

		n=1	n=2	n=3	n=4	n=5
**Cross**	$\bar{s}$/mS m^−1^	605.102	600.247	595.509	592.737	590.278
	$u_{r}\left(\bar{s}\right)$/%	0.162	0.114	0.122	0.131	0.135
	${\bar{s}}_{w}$/mS m^−1^	586.238	586.609	586.788	586.666	586.765
	$u_{r}\left({\bar{s}}_{w}\right)$/%	0.009	0.009	0.010	0.011	0.012
**Sphere**	$\bar{s}$/mS m^−1^	605.102	599.126	592.654	590.074	586.656
	$u_{r}\left(\bar{s}\right)$/%	0.162	0.112	0.120	0.136	0.147
	${\bar{s}}_{w}$/mS m^−1^	586.238	586.811	585.451	586.550	584.741
	$u_{r}\left({\bar{s}}_{w}\right)$/%	0.009	0.009	0.010	0.011	0.011
**Cube**	$\bar{s}$/mS m^−1^	600.452	592.570	586.605	582.032	578.445
	$u_{r}\left(\bar{s}\right)$/%	0.107	0.120	0.140	0.163	0.190
	${\bar{s}}_{w}$/mS m^−1^	586.239	585.582	585.287	582.176	580.104
	$u_{r}\left({\bar{s}}_{w}\right)$/%	0.009	0.010	0.011	0.013	0.017

## Data Availability

Data available in a publicly accessible repository: https://doi.org/10.5281/zenodo.4248879 (accessed on 13 February 2023).

## References

[1] Gulani V., Seiberlich N. (2020). Quantitative MRI: Rationale and challenges. Advances in Magnetic Resonance Technology and Applications.

[2] European Society of Radiology (ESR) (2013). ESR statement on the stepwise development of imaging biomarkers. Insights Imaging.

[3] Liu J., Wang Y., Katscher U., He B. (2017). Electrical properties tomography based on B1 maps in MRI: Principles, applications, and challenges. IEEE Trans. Biomed. Eng..

[4] Kim S.-Y., Shin J., Kim D.-H., Kim M.J., Kim E.-K., Moon H.J., Yoon J.H. (2016). Correlation between conductivity and prognostic factors in invasive breast cancer using magnetic resonance electric properties tomography (MREPT). Eur. Radiol..

[5] Tha K.K., Katscher U., Yamaguchi S., Stehning C., Terasaka S., Fujima N., Kudo K., Kazumata K., Yamamoto T., Van Cauteren M. (2018). Noninvasive electrical conductivity measurement by MRI: A test of its validity and the electrical conductivity characteristics of glioma. Eur. Radiol..

[6] Tha K.K., Kikuchi Y., Ishizaka K., Kamiyama T., Yoneyama M., Katscher U. (2021). Higher electrical conductivity of liver parenchyma in fibrotic patients: Noninvasive assessment by electric properties tomography. Magn. Reson. Imaging.

[7] Shu L., Böhm R., Katscher U., Jensen-Kondering U., Scholkmann F., LaManna J., Wolf U. (2022). Brain tissue conductivity in focal cerebral ischemia. Oxygen Transport to Tissue XLIII..

[8] Gavazzi S., van Lier A.L.H.M.W., Zachiu C., Jansen E., Lagendijk J.J.W., Stalpers L.J.A., Crezee H., Kok H.P. (2020). Advanced patient-specific hyperthermia treatment planning. Int. J. Hyperth..

[9] Katscher U., Kim D.-H., Seo J.K. (2013). Recent progress and future challenges in MR electric properties tomography. Comput. Math. Methods Med..

[10] Leijsen R., Brink W., van den Berg C., Webb A., Remis R. (2021). Electrical properties tomography: A methodological review. Diagnostics.

[11] Gabriel S., Lau R.W., Gabriel C. (1996). The dielectric properties of biological tissues: II. Measurements in the frequency range 10 Hz to 20 GHz. Phys. Med. Biol..

[12] Hoult D.I. (2000). The principle of reciprocity in signal strength calculations—A mathematical guide. Concepts Magn. Reson..

[13] Stollberger R., Wach P. (1996). Imaging of the active B1 field in vivo. Magn. Reson. Med..

[14] Yarnykh V.L. (2007). Actual flip-angle imaging in the pulsed steady state: A Method for rapid three-dimensional mapping of the transmitted radiofrequency field. Magn. Reson. Med..

[15] Sacolick L.I., Wiesinger F., Hancu I., Vogel M.W. (2010). B1 mapping by bloch-siegert shift. Magn. Reson. Med..

[16] Nehrke K., Börnert P. (2012). DREAM-a novel approach for robust, ultrafast, multislice *B*_1_ mapping. Magn. Reason. Med..

[17] Cloos M.A., Wiggins C., Wiggins G., Sodickson D. Plug and play parallel transmission at 7 and 9.4 Tesla based on principles from MR fingerprinting. Proceedings of the Joint Annual Meeting ISMRM-ESMRMB & ISMRT 31st Annual Meeting.

[18] Lee S.-K., Bulumulla S., Wiesinger F., Sacolick L., Sun W., Hancu I. (2015). Tissue Electrical property mapping from zero echo-time magnetic resonance imaging. IEEE Trans. Med. Imaging.

[19] Bucci O.M., Isernia T. (1997). Electromagnetic inverse scattering: Retrievable information and measurement strategies. Radio Sci..

[20] Coli V.L., Tournier P.-H., Dolean V., Kanfoud I.E., Pichot C., Migliaccio C., Blanc-Feraud L. (2019). Detection of simulated brain strokes using microwave tomography. IEEE J. Electromagn. RF Microw. Med. Biol..

[21] Dachena C., Fedeli A., Fanti A., Lodi M.B., Pastorino M., Randazzo A. (2021). Microwave imaging for the diagnosis of cervical diseases: A feasibility analysis. IEEE J. Electromagn. RF Microw. Med. Biol..

[22] Balidemaj E., van den Berg C.A.T., Trinks J., van Lier A.L.H.M.W., Nederveen A.J., Stalpers L.J.A., Crezee H., Remis R.F. (2015). CSI-EPT: A contrast source inversion approach for improved MRI-based electric properties tomography. IEEE Trans. Med. Imaging.

[23] Ammari H., Kwon H., Lee Y., Kang K., Seo J.K. (2015). Magnetic resonance-based reconstruction method of conductivity and permittivity distributions at the larmor frequency. Inverse Probl..

[24] Arduino A., Zilberti L., Chiampi M., Bottauscio O. (2017). CSI-EPT in presence of RF-shield for MR-coils. IEEE Trans. Med. Imaging.

[25] Rahimov A., Litman A., Ferrand G. (2017). MRI-based electric properties tomography with a quasi-newton approach. Inverse Probl..

[26] Hong R., Li S., Zhang J., Zhang Y., Liu N., Yu Z., Liu Q.H. (2017). 3-D MRI-based electrical properties tomography using the volume integral equation method. IEEE Trans. Microw. Theory Techn..

[27] Arduino A., Bottauscio O., Chiampi M., Zilberti L. (2018). Magnetic resonance-based imaging of human electric properties with phaseless contrast source inversion. Inverse Probl..

[28] Leijsen R.L., Brink W.M., van den Berg C.A.T., Webb A.G., Remis R.F. (2018). 3-D contrast source inversion-electrical properties tomography. IEEE Trans. Med. Imaging.

[29] Guo L., Jin J., Liu C., Liu F., Crozier S. (2018). An efficient integral-based method for three-dimensional MR-EPT and the calculation of the RF-coil-induced Bz field. IEEE Trans. Biomed. Eng..

[30] Bevacqua M.T., Bellizzi G.G., Crocco L., Isernia T. (2019). A method for quantitative imaging of electrical properties of human tissues from only amplitude electromagnetic data. Inverse Probl..

[31] Serralles J.E.C., Lattanzi R., Giannakopoulos I.I., Zhang B., Ianniello C., Cloos M.A., Polimeridis A.G., White J.K., Sodickson D.K., Daniel L. (2020). Noninvasive estimation of electrical properties from magnetic resonance measurements via global maxwell tomography and match regularization. IEEE Trans. Biomed. Eng..

[32] Giannakopoulos I.I., Serralles J.E.C., Daniel L., Sodickson D.K., Polimeridis A.G., White J.K., Lattanzi R. (2021). Magnetic-resonance-based electrical property mapping using global maxwell tomography with an 8-channel head coil at 7 Tesla: A simulation study. IEEE Trans. Biomed. Eng..

[33] Shin J., Kim M.J., Lee J., Nam Y., Kim M., Choi N., Kim S., Kim D.-H. (2015). Initial Study on in vivo conductivity mapping of breast cancer using MRI: In vivo conductivity mapping of breast cancer. J. Magn. Reson. Imaging.

[34] Voigt T., Katscher U., Doessel O. (2011). Quantitative Conductivity and permittivity imaging of the human brain using electric properties tomography: In vivo electric properties tomography. Magn. Reson. Med..

[35] Van Lier A.L.H.M.W., Brunner D.O., Pruessmann K.P., Klomp D.W.J., Luijten P.R., Lagendijk J.J.W., van den Berg C.A.T. (2012). *B*_1_^+^ phase mapping at 7 T and its application for in vivo electrical conductivity mapping: Electrical conductivity mapping. Magn. Reson. Med..

[36] Arduino A. (2021). EPTlib: An open-source extensible collection of electric properties tomography techniques. Appl. Sci..

[37] Stehning C., Voigt T.R., Katscher U. Real-time conductivity mapping using balanced SSFP and phase-based reconstruction. Proceedings of the 19th Scientific Meeting of the International Society of Magnetic Resonance in Medicine (ISMRM’11).

[38] Katscher U., Weiss S. (2022). Mapping electric bulk conductivity in the human heart. Magn. Reson. Med.

[39] Seo J.K., Kim M.O., Lee J., Choi N., Woo E.J., Kim H.J., Kwon O.I., Kim D.H. (2012). Error analysis of nonconstant admittivity for MR-based electric property imaging. IEEE Trans. Med. Imaging.

[40] Mandija S., Sbrizzi A., Katscher U., Luijten P.R., van den Berg C.A.T. (2018). Error analysis of helmholtz-based MR-electrical properties tomography: MR-electrical properties tomography reconstruction errors. Magn. Reson. Med..

[41] Cashmore M.T., McCann A.J., Wastling S.J., McGrath C., Thornton J., Hall M.G. (2021). Clinical quantitative MRI and the need for metrology. BJR.

[42] Lee S.-K., Bulumulla S., Hancu I. (2015). Theoretical investigation of random noise-limited signal-to-noise ratio in MR-based electrical properties tomography. IEEE Trans. Med. Imaging.

[43] Arduino A., Chiampi M., Pennecchi F., Zilberti L., Bottauscio O. (2017). Monte Carlo method for uncertainty propagation in magnetic resonance-based electric properties tomography. IEEE Trans. Magn..

[44] Schäfer J., Strimmer K. (2005). A shrinkage approach to large-scale covariance matrix estimation and implications for functional genomics. Stat. Appl. Genet. Mol. Biol..

[45] Opgen-Rhein R., Strimmer K. (2007). Accurate ranking of differentially expressed genes by a distribution-free shrinkage approach. Stat. Appl. Genet. Mol. Biol..

[46] Cox M.G., Eiø C., Mana G., Pennecchi F. (2006). The generalized weighted mean of correlated quantities. Metrologia.

[47] Stogryn A. (1971). Equations for calculating the dielectric constant of saline water (correspondence). IEEE Trans. Microw. Theory Techn..

[48] Voigt T., Homann H., Katscher U., Doessel O. (2012). Patient-individual local SAR determination: In vivo measurements and numerical validation: In vivo local sar measurement. Magn. Reson. Med..

[49] Savitzky A., Golay M.J.E. (1964). Smoothing and differentiation of data by simplified least squares procedures. Anal. Chem..

[50] Joint Committee for Guides in Metrology (JCGM) (2008). Evaluation of Measurement Data—Guide to the Expression of Uncertainty in Measurement.

[51] Joint Committee for Guides in Metrology (JCGM) (2011). Evaluation of Measurement Data—Supplement 2 to the “Guide to the Expression of Uncertainty in Measurement”—Models with Any Number of Output Quantities.

[52] Arduino A., Pennecchi F., Zilberti L., Katscher U., Cox M.G. (2020). EMUE-D5-3-EPTTissueCharacterization. https://zenodo.org/record/4248879#.Y-7nanYzaMo.

[53] Iyyakkunnel S., Bieri O. Conductivity mapping at 0.55 T with balanced steady state free precession. Proceedings of the Joint Workshop on MR Phase, Magnetic Susceptibility and Electrical Properties Mapping.

